# A Comprehensive Evaluation of the Burden of Heat-Related Illness and Death within the Florida Population

**DOI:** 10.3390/ijerph13060551

**Published:** 2016-05-31

**Authors:** Laurel Harduar Morano, Sharon Watkins, Kristina Kintziger

**Affiliations:** 1 Department of Epidemiology, Gillings School of Global Public Health, University of North Carolina at Chapel Hill, Chapel Hill, NC 27599, USA; 2 Florida Department of Health, Public Health Research Unit, Tallahassee, FL 32399, USA; shawatkins@pa.gov (S.W.); Kristina.Kintziger@flhealth.gov (K.K.); 3 Pennsylvania Department of Health, Bureau of Epidemiology, Harrisburg, PA 17120, USA

**Keywords:** heat, surveillance, morbidity, mortality, occupational, subtropical

## Abstract

The failure of the human body to thermoregulate can lead to severe outcomes (e.g., death) and lasting physiological damage. However, heat-related illness (HRI) is highly preventable via individual- and community-level modification. A thorough understanding of the burden is necessary for effective intervention. This paper describes the burden of severe HRI morbidity and mortality among residents of a humid subtropical climate. Work-related and non-work-related HRI emergency department (ED) visits, hospitalizations, and deaths among Florida residents during May to October (2005–2012) were examined. Sub-groups susceptible to HRI were identified. The age-adjusted rates/100,000 person-years for non-work-related HRI were 33.1 ED visits, 5.9 hospitalizations, and 0.2 deaths, while for work-related HRI/100,000 worker-years there were 8.5 ED visits, 1.1 hospitalizations, and 0.1 deaths. The rates of HRI varied by county, data source, and work-related status, with the highest rates observed in the panhandle and south central Florida. The sub-groups with the highest relative rates regardless of data source or work-relatedness were males, minorities, and rural residents. Those aged 15–35 years had the highest ED visit rates, while for non-work-related hospitalizations and deaths the rates increased with age. The results of this study can be used for targeted interventions and evaluating changes in the HRI burden over time.

## 1. Introduction

The term heat-related illness (HRI) captures a continuum of disorders that occur as the human body absorbs and creates more heat than can be dissipated [1,2,3]. As HRI progresses, single and multi-system failure occurs [4]. Timely medical intervention can prevent mild cases of HRI, such as heat edema, from becoming severe (e.g., heat stroke) and potentially resulting in death. However, even with medical intervention, severe HRI may have lasting effects, including neurological and organ damage and decreased heat tolerance, making an individual more susceptible to another HRI event [5,6,7]. Therefore, prevention of HRI is essential. Fortunately, HRI is highly preventable through individual- and community-level behavioral and structural modifications. For instance, an individual may move to a cooler environment (e.g., shade or air conditioned room), drink more fluids, or wear cooler clothing. A community may plant green space to reduce heat retention of impervious surfaces (e.g., pavement) or use more efficient building materials. Sports officials may hold outdoor events in the early morning or evening and employers may change the work/rest cycle to allow their employees time to dissipate excess body heat and reduce internal heat production. Modification may also include allowing the individual or community members (e.g., high school track team) approximately 2–3 weeks for acclimatization to occur prior to intense physical outdoor activity. To understand where and potentially which type of behavioral and structural modifications may have the largest effects on HRI prevention, characterization of HRI within the population and identification of groups with the highest (and lowest) burden is necessary.

The heat–health relationship (*i.e.*, heat-related outcomes and ambient outdoor heat) varies by latitude with those in the lower latitudes being better adapted to heat through behavioral and structural modification than those in higher latitudes. Even in areas well adapted to warm weather, individuals still succumb to HRI, especially individuals physically exerting themselves in the heat [8,9,10,11]. It is difficult to determine how HRI susceptibility factors previously identified in the literature vary by latitude and climate, as the majority of studies examining the heat–health relationship have been conducted in the northern United States (U.S.), Europe, and Australia [12,13,14,15,16,17,18,19,20]. Only a handful of studies globally have been conducted in tropical or humid subtropical areas such as the southeastern U.S. The tropical or humid subtropical climate may have an impact on the distribution of risk factors within the population. For instance, in a tropical or humid subtropical climate, sweating is an ineffective cooling mechanism potentially leading to an increased number of individuals working or playing outdoors succumbing to HRI compared with a dry or temperate climate [3,21].

The purpose of this paper is to describe the burden of severe HRI morbidity and mortality among Florida residents during the warm season. The results of these analyses identify populations appropriate for future investigations and targeted interventions. In addition, this analysis provides a baseline for future evaluations of interventions and for evaluating changes in the HRI burden over time. Finally, the results may be applicable to other areas of the southeastern U.S., and may provide a framework for exploring the HRI burden within other jurisdictions.

## 2. Materials and Methods

### 2.1. Data Sources

The analysis was restricted, unless otherwise mentioned, to Florida residents between May and October (2005–2012) [22]. Emergency department (ED) and hospital discharge data were obtained from the Florida Agency for Health Care Administration. The ED dataset contained those treated and released, while the hospital discharge dataset contained all admissions regardless of source. In order to estimate HRI onset, where available, the ED visit date was used [23]. Death certificate data were obtained from the Florida Department of Health (FDOH), Bureau of Vital Statistics. Again, in order to estimate the HRI onset, where available, both the date of injury and the date of death were analyzed [23]. Data sharing agreements were established with both data custodians. The agreements required that only summary data may be released by the authors.

The three datasets were not able to be linked, and therefore, had potentially overlapping cases. The overlap between the morbidity and mortality data was estimated via a variable in the morbidity data indicating if the patient died and a variable on the death certificate indicating if the death occurred in a hospital. Within the morbidity data, patients were included in both the ED and hospital discharge datasets if a patient was seen in the ED and transferred to another hospital (as opposed to the hospital connected to the ED). These transfer patients were identified via an ED discharge status indicating transfer to one of the following facilities: a short-term general hospital for inpatient care, children’s hospital, cancer center, Medicare certified long-term care hospital, psychiatric hospital or distinct unit, or critical access hospital.

In this analysis, individuals who sought treatment were released, and subsequently again sought treatment for the same event or who died would be counted twice within a single dataset (e.g., ED) or counted in multiple datasets. Unfortunately, it was not possible to identify these individuals. However, due to the disease course for HRI and effectiveness of treatment, it is assumed that these duplicate cases will be negligible.

### 2.2. Defining HRI

HRI was defined as the presence of an International Classification of Diseases, Ninth Revision, Clinical Modification (ICD-9-CM, morbidity) or Tenth Revision (ICD-10, mortality) code for the effects of heat and light (992–992.9/T67–T67.9) or an excessive heat external cause of injury (Ecode) (E900.0/X30, E900.1/W92, E900.9) [24,25,26]. All diagnosis/Ecode fields and underlying/contributing cause of death fields were used.

HRI cases were also stratified by work-relatedness. Workers may be at high risk for HRI, and susceptibility factors within this group may differ from the general population; however, characterization of HRI in this population is sparse [27]. In the morbidity datasets, work-relatedness was defined as expected payer equals workers’ compensation or the presence of the following ICD-9-CM Ecodes indicating that the injury occurred at work or a probable work-location: E000.0, E000.1, E800–E807 (4th digit = 0), E830–E838 (4th digit = 2 or 6), E840–E845 (4th digit = 2 or 8), E846, E849.1–E849.3 (Table 1) [25,28]. For mortality, if the death certificate variable *injury at work* was marked yes, then the death was considered work-related. Work-relatedness was restricted to those aged 16 years or older. All HRI cases not classified as work-related were classified as non-work-related.

### 2.3. Variable Selection

The following presents the parameterization used in this analysis for the individual-level factors identified based on a literature search.

Gender is associated with HRI morbidity and mortality, with men having a higher morbidity than women and the converse for mortality [18,29,30,31,32,33,34,35]. Gender was available in all datasets and modeled as binary (male/female).

Race and ethnicity have been observed to modify HRI rates. The differences are non-biological and suspected to be related to other factors (e.g., socio-economic status (SES), access to resources such as medical care, racial/ethnic distribution within occupation, or housing quality) [12,15,36,37,38,39,40]. In this study, race and Hispanic ethnicity will be examined in part to inform future analyses, which may include access to additional information related to factors such as SES, access to resources, or racial/ethnic distribution within outdoor occupations. Race was categorized as White, Black or African American, and other, while ethnicity was categorized as Hispanic or non-Hispanic.

The elderly [41,42,43,44] and very young (<5 years) [18,19] have been shown to have a high risk of HRI. Teenagers and young adults may also be at increased risk of HRI due to participation in athletics or high-risk occupational activities [16,32,45]. Age at time of visit, admission, or death was calculated by the data custodians and included in each dataset. For this analysis, age was grouped into 5-year age categories (0–4, 5–9, …, 30–34, 35–44, …, 75–79, 80–84, 85+).

Rural and urban populations may have different heat exposure and may react or adapt to heat differently because of differences in available resources (e.g., medical care, air conditioning usage, or outdoor activities) [46,47,48,49]. For each dataset, county of residence was used to determine rural or urban status. Rural and urban counties were defined in accordance with the definition provided by the FDOH Office of Rural Health based on the 2000 census (*i.e.*, rural counties equal a density of less than 100 persons per square mile) [50].

An individual’s SES may affect their ability to prevent an HRI outcome (e.g., lack of air conditioning or poor housing conditions) or reduce the severity of the outcome (e.g., access to medical care). Within the morbidity data, low SES was assessed using the potential payer field. Those records which had Medicaid, Kidcare (health insurance for low-income children age 0–18 provided by the state of Florida), or self-pay/charity listed as the payer were classified as low SES (*i.e.*, socially disadvantaged). A variable for assessing SES was not available in the mortality data. Note that the SES analysis was not stratified by work-related status, as the morbidity data only has a single payer code and part of the work-related definition is payer equal to worker’s compensation. Instead, all analysis related to SES was based on all HRI ED visits or hospitalizations.

As the thermoregulatory system fails, it affects multiple organs and systems throughout the body [4,51,52]. Further, individuals with chronic diseases or prior conditions are at higher risk of adverse HRI effects due to the increased stress caused by the thermoregulation process on already impaired organs and systems. For this analysis, among HRI cases, additional outcomes with consistent evidence of a positive relationship with heat and a hypothesized biological mechanism [2,4,18,20,51,53,54,55,56,57,58,59] were examined: cardiovascular disease (ICD-9-CM codes: 390–429, 440–448; ICD-10 codes: I00–I51, I70–I79), cerebrovascular disease (ICD-9-CM codes: 430–438; ICD-10 codes: I60–I69), respiratory disease (ICD-9-CM codes: 460–519; ICD-10 codes: J00–J99), renal disease (ICD-9-CM codes: 580–589; ICD-10 codes: N00–N07, N17–N19, N25–N27), diabetes mellitus (ICD-9-CM code: 250; ICD-10 codes: E10–E11, E13–E14) and injuries (ICD-9-CM codes: 800–904 and 910–959; ICD-10 codes: S00–T35). A direct biological mechanism is not present for injuries, however, the symptoms of HRI (e.g., neurological impairment such as dizziness) may lead to injury (e.g., a worker falls from scaffolding) [18,25,60,61,62,63].

Part of the HRI prevention message is an awareness of the signs and symptoms of HRI so that individuals may seek immediate treatment to prevent more severe outcomes [64,65]. Length of stay (LOS) within the hospital is a simple and easily calculable measurement that can, to some extent, represent severity of outcome and effectiveness of treatment (*i.e.*, shorter stay equals less severe outcome or more effective treatment). LOS was calculated in this study to provide a baseline estimate of HRI to aid in the understanding of the effectiveness of HRI awareness messaging. LOS was the difference between the date of hospital discharge and the date of hospital admission or the date of ED visit for those admitted through the ED. For decedents who were admitted to the hospital prior to death, LOS was calculated as the date of death minus the date of injury.

### 2.4. Analysis

Crude and, where appropriate, age-adjusted rates and standard errors (SE) were calculated for Florida overall and stratified by county or by the susceptibility factors of interest. The numerator was HRI ED visits, hospitalizations, or deaths. The denominator for the Florida population and civilian workforce was obtained from the Florida Legislature’s Office of Economic and Demographic Research and the Current Population Survey (CPS), respectively. The civilian workforce was enumerated as the number of civilian workers aged 16 years or older categorized as employed at work or employed absent. CPS county-specific estimates of the workforce were not available; therefore, for work-related county-specific analyses, the American Community Survey (ACS) 5-year estimates (2005–2009 and 2008–2012) were used. A limitation of using the 5-year ACS estimates is the assumption of a stable population. As the Florida population has increased over time, the 2008–2012 ACS was used for the years 2010–2012. Each yearly estimate was divided by two to produce the person-time at risk for May–October. Age-adjusted rates were standardized to the 2000 U.S. standard population using direct standardization. The crude rate ratios (RR) and corresponding 95% confidence intervals (CI) were calculated to compare rates of HRI by the aforementioned susceptibility factors (e.g., crude HRI rate in males divided by crude HRI rate in the referent group, females). Calculated measures of variance (e.g., 95% CI or SE) were used to indicate the stability of the rate or RR estimate.

The death certificate contains text fields with information on how the death occurred and the usual occupation of the decedent. The former is completed by the medical certifier and required for all deaths with an injury (e.g., HRI) or poisoning, while the latter is completed by the funeral director. These fields provided information related to the specific activity or situation of the decedent that contributed to the HRI death. These fields were reviewed and manually summarized (*i.e.*, codes created) into categories.

Institutional Review Board approval was obtained from the University of North Carolina (Study number: 14-2246). A waiver of informed consent was given, as this was a retrospective study using secondary data and data are only displayed as summary statistics. The study was also submitted to the FDOH Institutional Review Board, and it was determined that the study fell within the purview of public health practice and surveillance activities.

## 3. Results

### 3.1. HRI Seasonality

**Figure 1:**
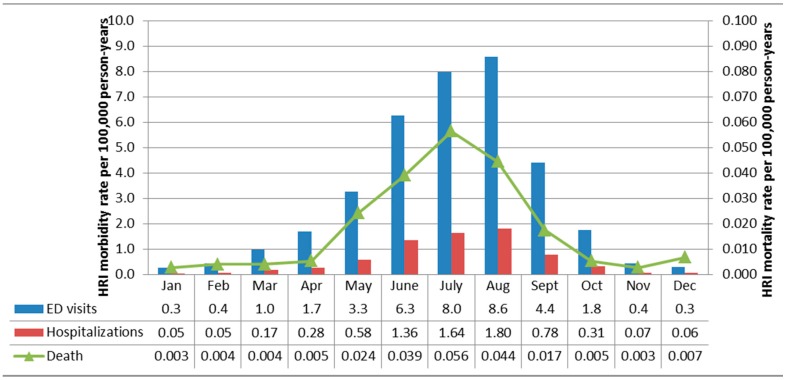
Non-work-related HRI morbidity and mortality rates for Florida residents stratified by month and data source, 2005–2012 (Total cases: ED visits = 27,028; Hospitalizations = 5324; Deaths = 158). All rates are per 100,000 person-years. Note that the morbidity rates (left y-axis) are two orders of magnitude larger than the mortality rates (right y-axis).

**Figure 2:**
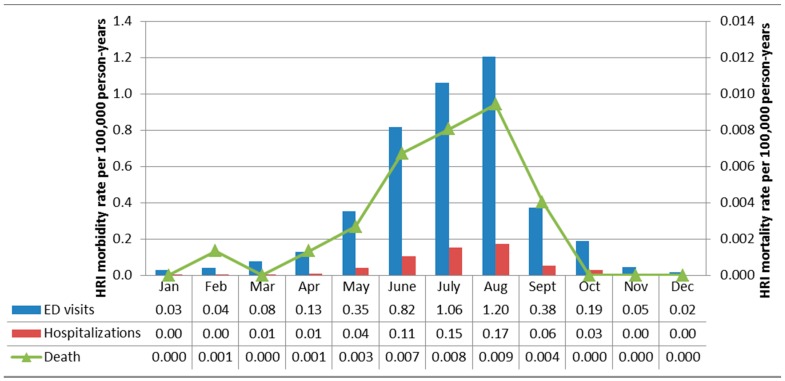
Work-related HRI morbidity and mortality rates for Florida residents stratified by month and data source, 2005–2012 (Total cases: ED visits = 3234; Hospitalizations = 432; Deaths = 25). All rates are per 100,000 person-years. Note that the morbidity rates (left y-axis) are two orders of magnitude larger than the mortality rates (right y-axis).

Using annual data from the entire study period (2005–2012), among Florida residents, the majority of non-work-related HRI ED visits (83.9%; *n =* 22,669), hospitalizations (86.1%; *n =* 4582), and deaths (85.4%; *n =* 135) were observed between May and September (Figure 1). The largest proportion of non-work-related ED visits (23.6%; *n =* 6377) and hospitalizations (25.1%; *n =* 1338) occurred in August while the largest proportion of non-work-related deaths, 26.6% (*n =* 42), occurred in July. A similar distribution was observed for work-related HRI, although the summer peak was slightly higher with 87.8 (*n =* 2838), 91.2 (*n =* 394), and 92.0% (*n =* 23) of ED visits, hospitalizations, and deaths occurring between May and September (Figure 2). The largest proportion of work-related HRI morbidity and mortality occurred in August (ED visits: *n =* 896, 27.7%; hospitalizations: *n =* 130, 30.1%; deaths: *n =* 7, 28.0%).

### 3.2. HRI Occurance

Among Florida residents, during the Florida warm season (May–October) for 2005–2012, there were 23,981 non-work-related HRI cases treated in the ED, 4816 HRI hospitalizations, and 139 HRI deaths. These cases accounted for 0.10% of all-cause warm season non-work-related ED visits, 0.05% of non-work-related hospitalizations, and 0.02% of non-work-related deaths. Among work-related HRI cases, there were 2979 cases treated in the ED, 415 hospitalizations, and 23 deaths. The work-related HRI cases accounted for 0.66%, 0.98%, and 2.3% of all-cause work-related ED visits, hospitalizations, and deaths during the warm season.

### 3.3. Emergency Department Visits

**Figure 3:**
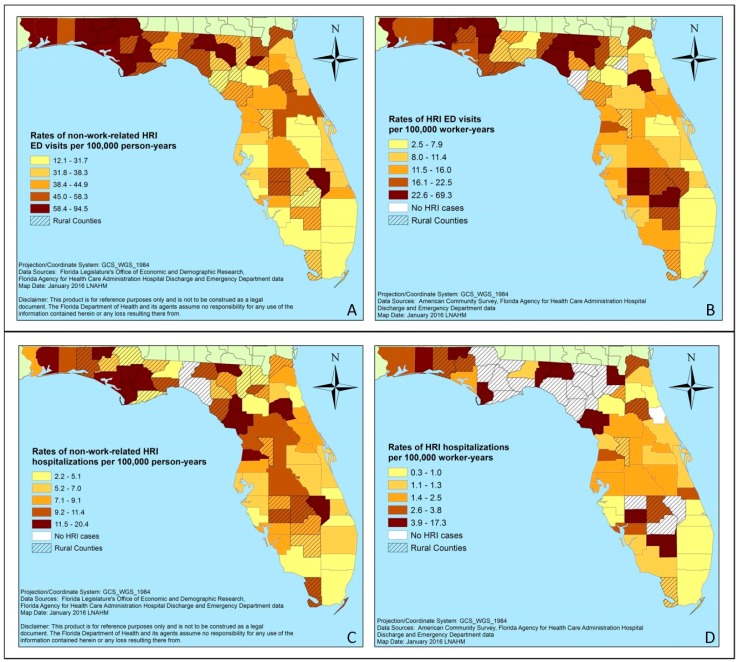
County-specific rates of HRI ED visits and hospitalizations among Florida residents during the warm season (2005–2012): (**A**) non-work-related HRI ED visits rates per 100,000 person-years; (**B**) work-related HRI ED visits rates per 100,000 worker-years; (**C**) non-work-related HRI hospitalizations rates per 100,000 person-years; (**D**) work-related HRI hospitalizations rates per 100,000 worker-years.

During the warm season of the eight-year study period, the age-adjusted rate for non-work related HRI ED visits among Florida residents was 33.11 visits per 100,000 person-years (SE = 2.17), while the age-adjusted rate for work-related HRI ED visits was 8.46 visits per 100,000 worker-years (SE = 1.40). Among those treated in the ED but not admitted to the hospital, there were 15 non-work related HRI deaths and 1 work-related HRI death. The highest rates of HRI ED visits were found in the Florida panhandle (Figure 3).

**Figure 4:**
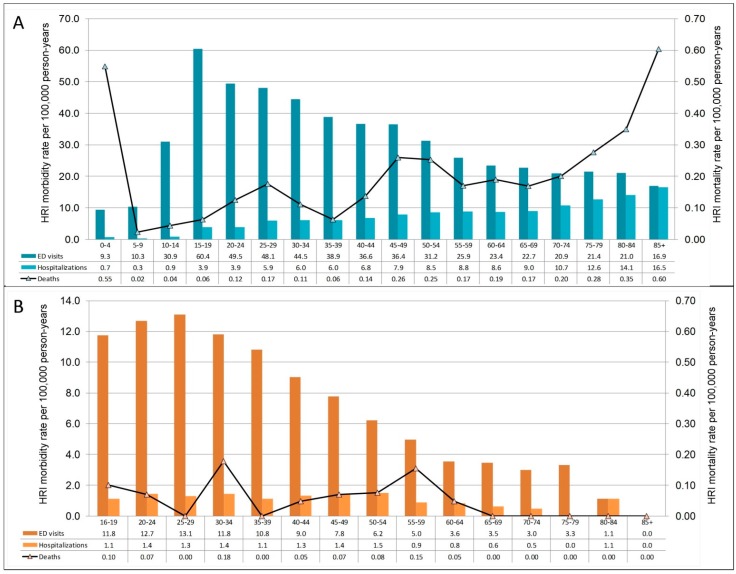
Age-specific rates of *non-work-related* (**A**) and *work-related* (**B**) HRI identified among Florida residents (2005–2012). All rates are per 100,000 person years. In panel (**A**), the non-work-related morbidity rates (left y-axis) are two orders of magnitude larger than the non-work-related mortality rates (right y-axis). In panel (**B**), the age distribution for work-related HRI starts at age 16, and the morbidity rates are approximately an order of magnitude larger than the mortality rates.

Demographic non-work-related and work-related HRI ED visit characteristics can be found in Table 2 and Table 3, respectively. The rate of HRI ED visits was higher among males than females (non-work-related: RR = 2.77; work-related: RR = 5.91), for minorities (Blacks and others) compared to Whites (non-work-related: Blacks RR = 1.43, others RR = 1.14; work-related: Blacks RR = 1.18, others RR = 1.27), and for rural areas *versus* non-rural areas (non-work-related: RR = 1.62; work-related: RR = 2.51). Rates were lower for Hispanics compared with non-Hispanics (non-work-related: RR = 0.46; work-related: RR = 0.56). The rate of non-work-related HRI ED visits by age group was highest for those aged 15–19 years (60.41 per 100,000 person-years) and decreased as age increased (Figure 4A). The lowest non-work-related HRI ED rate is among children less than 10 years of age (9.83 per 100,000 person-years). Work-related HRI ED rates were highest among those under the age of 35 years (12.46 per 100,000 worker-years) and lowest for those aged 70 years or older (2.69 per 100,000 worker-years) (Figure 4B).

**Figure 5:**
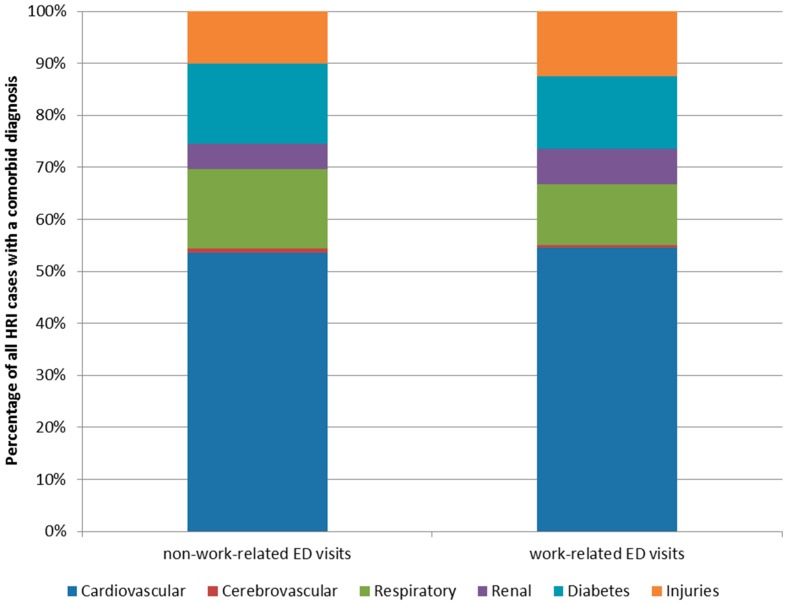
Distribution of co-morbid outcomes among Florida residents identified with HRI in the ED, stratified by work-related status (2005–2012).

Among non-work-related HRI ED visits, 28.3% (*n =* 6789) had a code for one or more of the selected co-morbid conditions; while for work-related cases, 24.8% had a co-morbid code (*n =* 738). Only 25.1% and 4.5% of co-morbid non-work-related and work-related HRI ED visits, respectively, were among individuals age 65 years or older. The most frequent co-morbid codes for non-work-related HRI cases were cardiovascular disease (67.2%; *n =* 4565 (age ≥ 65: 33.1% = 1512/4565)), diabetes (19.4%; *n =* 1319 (age ≥ 65: 30.7% = 405/1319)), and respiratory disease (19.2%; *n =* 1302 (age ≥ 65: 12.4% = 162/1302)) (Figure 5). While for work-related HRI, the most frequent co-morbid codes were cardiovascular disease (64.7%; *n =* 479 (age ≥ 65: 6.5% = 31/479)), diabetes (16.5%; *n* = 122 (age ≥ 65: 4.1% = 5/122)), and injuries (14.9%; *n =* 110 (age ≥ 65: *n* < 5)) (Figure 5).

Forty-four percent (*n =* 11,981) of all HRI ED cases were identified as low SES according to the payer codes, while 54.4% (*n* = 12,847,894) of total all-cause ED visits (*n =* 23,601,926) were identified as low SES.

### 3.4. Hospitalizations

During the study period, the age-adjusted rates for non-work-related and work-related HRI hospitalizations were 5.88 hospitalizations per 100,000 person-years (SE = 0.87) and 1.12 hospitalizations per 100,000 worker-years (SE = 0.51), respectively. During the study period, there was an average of 14.6 non-work-related HRI ED visits per year (*n =* 117) where the patient was transferred to a hospital not connected to the visited ED and potentially counted in both morbidity datasets. Additionally, there were less than five work-related HRI cases where the patient was transferred to a hospital not connected to the visited ED. Fifty-six non-work-related deaths and 3 work-related deaths were identified among those hospitalized with HRI. The counties with the highest rates of non-work-related and work-related HRI hospitalizations were scattered across the northern and middle portion of the state (Figure 3). The mean LOS for non-work-related hospitalizations was 3.1 days (SE = 0.07; Median *=* 2; Range = 0–116), while the mean LOS for work-related hospitalizations was slightly lower at 2.5 days (SE = 0.19; Median *=* 2; Range = 0–53).

The demographic patterns for hospitalizations were the same as ED visits except for the age-group distribution (Table 2 and Table 3). Hospitalizations for non-work-related HRI increased as age increased, with the lowest rate for ages 0–14 (0.65 per 100,000 person-years) and the highest rate for those 75 years or older (14.17 per 100,000 person-years) (Figure 4A). The highest rates of work-related HRI were for those aged 45 to 54 years (1.46 per 100,000 worker-years) and lowest for those 70 years of age or older (0.38 per 100,000 worker-years) (Figure 4B).

**Figure 6:**
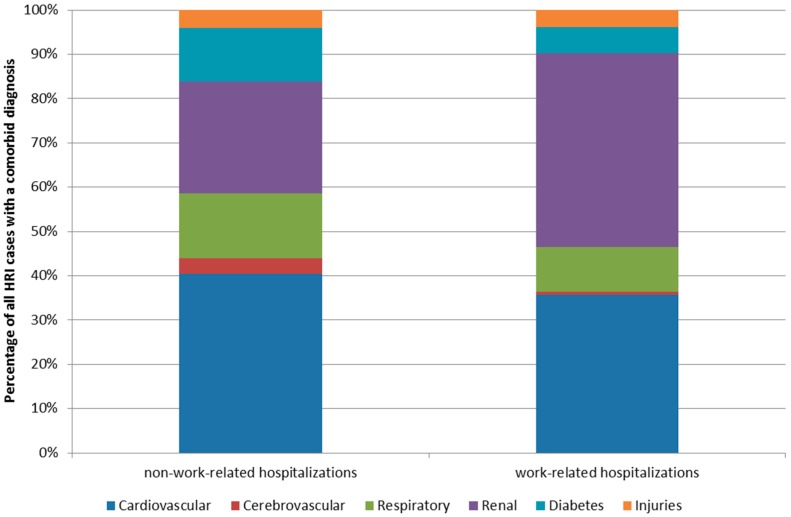
Distribution of co-morbid outcomes among Florida residents identified with HRI in the hospital, stratified by work-related status (2005–2012).

The proportion of HRI hospitalizations with a code for one or more of the selected co-morbid conditions was slightly higher for non-work-related (80.8%; *n =* 3893) than work-related cases (72.2%; *n =* 302). For both non-work-related and work-related HRI hospitalizations, the most frequent co-morbid codes were for cardiovascular disease (non-work-related: 69.1%, *n* = 2691; work-related: 53.5%, *n =* 160) and renal outcomes (non-work-related: 44.5%, *n =* 1693; work-related: 64.9% *n* =196) (Figure 6). A little under a third (*n =* 3134) of co-morbid non-work-related HRI hospitalizations were among individuals age 65 years or older (cardiovascular: 48.3% = 1200/2691; renal: 20.3% = 343/1693). While for work-related HRI hospitalizations, only 3.9% (*n =* 41) of HRI hospitalizations were for those age 65 years or older (cardiovascular: 4.4% = 7/160; renal: *n* < 5).

The indicator of low SES was recorded in 34% (*n =* 1754) of all HRI hospitalizations and 28.6% (*n =* 2,836,156) of total all-cause hospitalizations (*n =* 9,907,258).

### 3.5. Deaths

The age-adjusted rate of HRI death during the warm season of the eight-year study period was 0.17 per 100,000 person-years (SE = 0.15) for non-work-related HRI and 0.06 per 100,000 worker-years (SE = 0.11) for work-related HRI. According to the death certificate, 60% (*n*: non-work-related = 80; work-related = 17) of all individuals who died of (or with) HRI were taken to the hospital or ED prior to death. Among the 36 decedents admitted to the hospital, as recorded in the death certificate, prior to death with an available date of HRI onset, the average LOS was 3.6 days for non-work-related HRI deaths (*n =* 29; SE = 0.99, Median *=* 1, Range = 0–22) and 9.3 for work-related HRI deaths (*n =* 6; SE = 6.08; Median *=* 3.5; Range = 0–39). The number of deaths per county was too small to provide stable county-specific rates.

Table 2 contains demographic characteristics for non-work-related HRI deaths. The results were similar to the non-work-related morbidity results except for age. The rate of non-work-related deaths was highest for the very young (age < 5: Rate = 0.55/100,000 person-years) and the elderly (age 75+: Rate = 0.39/100,000 person-years), followed by those aged 45–54 (Rate = 0.26/100,000 person-years) (Figure 4A).

The demographic characteristics for work-related HRI deaths can be found in Table 3. Unlike work-related HRI morbidity, the rate of work-related HRI deaths was higher among Hispanics than among non-Hispanics (RR = 2.35; 95% CI = 1.02, 5.43). Age-specific rates were highest for workers aged 30–34 years (0.18 per 100,000 worker-years) and 55–59 years (0.15 per 100,000 worker-years) (Figure 4B). Among decedents with work-related HRI, only half (*n =* 11) of the death certificates had the usual industry/occupation listed, of which all were outdoor workers.

Among non-work-related and work-related HRI deaths, 33.7% (*n =* 55) and 2.5% (*n =* 4) of deaths, respectively, had a code for one or more of the selected co-morbid conditions. Twenty-seven of the non-work-related HRI deaths and none of the work-related HRI deaths with a co-morbid condition were among those age 65 years or older. The most common condition was cardiovascular disease (non-work-related: 81.8%, *n =* 45 (age ≥ 65: 55.6% = 25/45); work-related = 100%, *n =* 4).

There were 137 (84.6% (n: non-work-related = 114; work-related = 23)) death certificate records which contained text information on how the death occurred. Among those HRI deaths with situational information, 20.4% (*n*: non-work-related = 13; work-related = 15) of the notes explicitly mentioned the decedent exerting themselves in hot weather, 10.9% (*n*: non-work-related = 14; work-related = 1) were related to alcohol intoxication or illicit drug use, and 27.7% (*n*: non-work-related = 37; work-related = 1) were due to being trapped in a car. For those died trapped in a car, 57.9% (*n =* 22) were under the age of 5 years.

## 4. Discussion

This paper provides an overview of the burden of HRI resulting in a death or provision of medical services in an ED or hospital among Florida residents. This is one of a small number of studies that have looked at the burden of HRI and the first study within the southeastern U.S. to use multiple data sources (*i.e.*, ED, hospitalization, and death certificates) for this evaluation [25,26,32,38,66,67,68,69,70,71,72,73,74]. Most studies have examined risk factors for heat-related outcomes (which include but are not limited to HRI, all-cause morbidity/mortality, cardiovascular disease, or respiratory disease) in relation to high temperatures or heat-waves [12,18,19,20]. However, HRI can occur outside heat waves and extreme temperatures. Using multiple data sources provides a more complete understanding of the HRI burden, as well as a better baseline for assessing changes in the HRI distribution within the population.

HRI burden differed geographically throughout the state. The lowest rates of HRI morbidity were observed in southern Florida counties, while the highest rates of HRI morbidity were observed in counties in the panhandle, or in the northern part of the state. Average summer temperatures are higher and more variable in the northern part of the state compared to average summer temperatures in the southern part of the state [75]. High rates of work-related HRI were also observed in south central Florida, an area with a large proportion of citrus agriculture, which is labor intensive [76,77]. The highest HRI morbidity counts were found in counties with urban centers: Broward, Hillsborough, Orange, Palm Beach, Pinellas, and Miami-Dade (Figure S1). Counties with the highest rates of HRI morbidity differed by data source, while counties with the highest counts did not.

Within this study, higher HRI morbidity and mortality rates were also observed for rural compared to urban counties. This result has been observed in prior studies [26,78]. Rural-urban differences in the burden of HRI may be explained by differences in the distribution of factors such as disparities in socio-economic status or access to health care, greater (or different) occupational risk factors (e.g., agricultural work), or differences in the proportion and type of impervious surfaces (e.g., higher humidity due to more vegetation in rural agricultural areas) [46,47,48,78,79]. For instance, a study of HRI ED visits in North Carolina (2007–2012) found that for rural zip codes, HRI decreased as the percentage of impervious surfaces and developed land increased [80]. However, in urban areas, impervious surfaces absorb heat contributing to an urban heat island effect and were associated with increased morbidity and mortality [81]. Further, a study by Hajat *et al.* classified rural/urban census output areas (*i.e.*, the smallest geographical unit used in the 2001 United Kingdom census areas with an average population size of 300) in England and Wales and found that rural areas with the highest level of social deprivation (*i.e.*, lowest SES) had the highest relative risk of death [82]. (Of note, according to the figures presented by Hajat *et al.*, the confidence intervals were tighter around the urban than the rural estimates, indicating greater variability for the rural results.)

The distribution of rates of HRI by age group differed for ED visits (treated and released), hospitalizations, and deaths. Within the ED data, the highest rates of HRI were among teenagers and young adults, regardless of work-related status. The highest hospitalization rates for non-work-related HRI were among the elderly. The highest HRI death rates were for the elderly and those under age five. This non-work-related HRI age-group distribution is similar to what has been shown in studies that have included age at a finer resolution than 15–64 years (morbidity [26,32,66,72,73]; mortality [74,83,84,85]). Both the elderly and very young children have a decreased ability to thermoregulate due to biologic effects (e.g., aging or underdevelopment) and decreased ability (or inability) to employ behavioral modifications [18,19,41,42,43,44]. Further, the prevalence of co-morbid conditions, which put individuals at increased risk of HRI, increases with age. In this analysis, comparing HRI ED visits and hospitalizations, the proportion of selected co-morbid conditions among HRI hospitalizations was greater than among HRI ED visits.

Heat stroke can be divided into two categories: classical and exertional. Classical heat stroke typically develops over days and occurs in hot environments among those whose thermoregulatory system is compromised [2,3,86]. Exertional heat stroke, on the other hand, can develop within hours often in young, healthy individuals under conditions of strenuous activity, usually in hot humid weather [2,3]. Those with compromised thermoregulatory systems are also susceptible when involved in strenuous activities. The data used for the present analysis do not include variables to identify classical and exertional heat stroke. Although it was not possible to distinguish between the two heat stroke subtypes, 43.5% and 64.9% of all non-work-related and work-related HRI hospitalizations, respectively, had a renal diagnosis. Acute renal failure is a common symptom of exertional heat stroke [2,3,4,86]. The higher proportion of cases with a renal outcome among work-related HRI hospitalizations may be related to individuals exerting themselves in the heat as part of their job duties. Further, of those HRI deaths with a situational text note on the death certificate, 11.4% and 65.2% of non-work-related and work-related deaths, respectively, explicitly indicated exertion during hot weather. A potentially higher proportion of exertional heat stroke in the Florida population (*versus* classical heat stroke) may have implications for how HRI prevention is implemented, as well as how HRI is modeled when examining the heat–health relationship or estimating projections of future burden. For instance, it may be more effective to target schools, athletic events, or outdoor workers for prevention programs as opposed to targeting the elderly or isolated individuals. It may also be more appropriate to model heat as a continuous exposure over time as opposed to binary, heat wave *versus* non-heat waves.

Among Florida residents, men have higher rates of both HRI morbidity and mortality than women, regardless of work-related status. For morbidity, this result reflects what has been observed in the literature [29,30,31,32]. Researchers have suggested that this difference may reflect the activities men (*versus* women) are involved in [32]. For instance, Kerr and colleagues used the National High School Sports-Related Injury Surveillance System data (2005/2006–2010/2011) to examine HRI among high school athletes [16]. They found that the majority of HRI (87.7%) occurred among boys; however, when football players were removed, boys accounted for 50.9% of events [16]. Occupationally speaking, males are more likely to work in jobs with greater risk of HRI, such as construction or fire protection [45,87]. This may also explain the larger gender-specific relative difference observed for work-related compared to non-work-related HRI. The majority of the literature examining heat-related mortality and gender found that either mortality was greater for women than for men [18,33,34,35] or no association [53,88]. Men and women have different underlying HRI risk profiles. For instance, unmarried elderly men tend to be at higher risk of HRI due to social isolation [15,43]. Women have a higher proportion of chronic conditions than men due to living longer, and this places women at higher risk of HRI than men. The distribution of these underlying HRI risk profiles within each population will impact whether gender differences in HRI rates are observed or not observed [12,15]. Additionally, the gender differences observed in the literature may be a reflection of the outcome used [88,89]. Many studies use all-cause mortality or a cause-specific outcome (e.g., all cardiovascular or all respiratory disease) when examining the heat–health relationship [18]. However, in the few studies that looked specifically at HRI mortality, men had a higher risk of HRI than women [38,67,84,90]. Further, an analysis of heat-related mortality among U.S. workers (2000–2010) also saw much higher rates of HRI among male workers than among female workers [27].

Full understanding of the racial and ethnic differences observed within Florida requires further exploration. The lower rate of non-work-related HRI morbidity and mortality among Hispanics compared with non-Hispanic Whites may be related to the unique distribution of Hispanic origin groups within the state (discussed below). As such, this lower rate observed in this study may not be reflective of the HRI burden among all Hispanic origin groups within Florida. Further, the similar and higher work-related HRI hospitalization and death rates, respectively, among Hispanics compared to non-Hispanics may be due to susceptibility factors unique to the sub-population employed in high HRI risk occupations. These susceptibility factors may include language barriers, management style, and cultural identity [91,92]. South Florida has a large Hispanic population; however, this region also has some of the lowest rates of HRI. South Florida is predominantly Hispanic (64.5% of population in Miami-Dade County) with the plurality being of Cuban origin (34.3%) [93,94]. With such a majority of Hispanics in South Florida, Hispanics may represent a large percentage of nearly every industry, occupation, and job class. Further, as Klinenberg noted in relation to the 1995 Chicago heat-wave, the social and spatial composition of Hispanic communities including high population density and vibrant/active public and retail spaces may have contributed to the lower mortality rates among Hispanics [15]. In North Florida, Hispanics comprise a small portion of the population (5% of population in the panhandle (Panhandle = Bay County, Calhoun County, Escambia County, Franklin County, Gadsden County, Gulf County, Holmes County, Jackson County, Jefferson County, Leon County, Liberty County, Madison County, Okaloosa County, Santa Rosa County, Taylor County, Wakulla County, Walton County, Washington County)) and are primarily of non-Cuban descent (e.g., 39% of panhandle Hispanics are of Mexican origin), and the vast majority of these individuals are employed in the service or construction/extraction occupations, which are major occupation groups at high risk for HRI [45,94,95] (CPS 2005–2011). In addition, there may be other (protective) factors involved in residing in South Florida, such as cooling ocean breezes or access to medical care [96].

Few researchers have considered the relationship between heat and injuries when evaluating heat-related outcomes [62,85,97,98], and injuries are often excluded when the relationship between heat and all-cause mortality or morbidity is analyzed [18,60,99,100]. As HRI progresses, it affects the neurological system, potentially resulting in decreased cognitive function (including short-term memory) and motor skills [17,101]. Within this analysis, injuries were identified as a potentially important co-morbid condition for HRI, especially for work-related cases. Fifteen percent of work-related and 13% of non-work-related HRI ED visits (treated and released) with a selected co-morbid condition had a diagnosis code indicating an injury. Injury diagnoses were also present among HRI hospitalizations and non-work-related HRI deaths, indicating the potential need for inclusion of injuries in further research and interventions related to the prevention of HRI. This may be especially true for workers at risk of HRI who work in potentially precarious situations (e.g., on scaffolding) or with potentially dangerous equipment (e.g., nail gun).

### Limitations

The present analysis does not capture the full burden of HRI in Florida. Individuals seeking treatment at medical facilities outside of an ED or hospital (e.g., doctor’s office or urgent care) or individuals not seeking care but remaining alive are not included in this analysis. Future research, with additional data sources, will be required to estimate the full burden of HRI in Florida and to characterize the distribution of susceptibility factors among these additional cases. Although this limitation must frame the results presented here, the present analysis characterizes the burden of HRI as severe enough to warrant medical attention in the ED or hospital or that results in death. Prevention and interventions targeted to those groups (e.g., males) with the largest burden of severe HRI will reduce the overall burden of HRI, as well as reduce overall costs to the health care system. Additionally, this analysis was limited to Florida residents because denominator data were not available to estimate the non-resident population. However, Florida industries employ thousands of out-of-state workers and the large tourism industry brings millions of non-residents (tourists) to the state [102,103]. These individuals may not be acclimatized to the Florida climate and may be at increased risk of HRI. Although not part of the analyses, during the study period there were 2493 non-work-related HRI ED visits, 308 HRI hospitalizations, and 10 deaths among non-residents, as well as an additional 128 work-related ED visits, 25 hospitalizations, and 4 deaths among non-residents. Future research will also be required to characterize the distribution of susceptibility factors among non-residents.

The majority of work-related HRI cases were identified by workers’ compensation as the expected payer source (ED visits = 78.1%; Hospitalizations = 69.2%). However, there are many barriers to a worker’s ability to access workers’ compensation, and many of those at high risk for work-related HRI may not qualify for workers’ compensation or may be unaware of its availability [92]. While the use of Ecodes for classifying work-relatedness helps identify those individuals not captured by workers’ compensation, the Ecodes, especially the location codes, may incorrectly classify non-work-related cases as work-related. As work-relatedness is highly under-identified [104,105,106], classifying non-work-related cases as work-related via Ecodes is assumed to result in a smaller amount of bias than incorrectly classifying work-related cases as non-work-related.

A further limitation of these analyses is the reliance on diagnosis codes. Assignment of ICD-9-CM/ICD-10 codes and their usage (or non-usage) varies by facility and geographic location [19,107,108]. No uniform guidelines exist to inform the assignment of a HRI. As a result, HRIs are often recorded in the medical or death record as the condition that arises from response to the stress of the thermoregulatory process (e.g., acute renal failure, acute respiratory distress syndrome, or myocardial injury) or the pre-existing condition that may have been exacerbated by the stress of the thermoregulatory process (e.g., cardiovascular disease, renal failure, or diabetes) [4,19,51,52,109]. This leads to a potential under-reporting of HRI. It is unclear if the potential under-reporting of HRI is differential with respect to the susceptibility factors examined here. Non-differential under-reporting of HRI within susceptibility factors would increase the rates but would not change the ratio of observed differences.

The unit of analysis for this study was the county of residence, the smallest geographic unit available for which Florida population estimates could be obtained from the Florida Legislature’s Office of Economic and Demographic Research. These population estimates provide a reasonably accurate estimation of person-time which do not require the assumption of a constant population during the study time period or the interpolation of decennial census data. The data from the Florida Legislature’s Office of Economic and Demographic Research combines multiple data sources to estimate how the Florida population demographics changes from year to year [110]. Unfortunately, summarizing the data via county can obscure within county differences, which may be large. For instance, Orange County includes both a large urban area (*i.e.*, Orlando) and rural agricultural areas; for this analysis Orange County was classified as urban. In this situation, if the majority of the HRI cases in an urban classified county were in the rural area (*i.e.*, misclassified as urban), then the rural/urban differences would be larger than originally estimated. If the reverse were true, where the majority of HRI cases were in the urban area of an urban classified county (*i.e.*, correctly classified as urban), then the rural/urban results would not change. However, when targeting HRI prevention to rural areas, it is important to know if rural areas within urban counties would benefit from prevention programs or if it is only rural areas within rural counties that would benefit. Analysis of HRI and various susceptibility factors within smaller geographic areas could provide further information useful in guiding the use of limited resources to prevent and reduce HRI. However, county-specific rates can be useful for evaluating reductions in the overall burden of HRI.

## 5. Conclusions

This study describes the burden of HRI among Florida residents using a combination of three data sources: ED, hospital discharge, and death certificates. These three datasets are not often used collectively within the heat-health literature. However, using this information in aggregate provides a more complete picture of the burden of HRI requiring medical attention. Regional and sub-population differences were observed, reflective of Florida’s diverse climate and population. The highest rates and counts of HRI were observed in males, those living in rural counties, and residents aged 15–35 years. HRI is highly preventable with behavioral modifications and community-level interventions. Therefore, interventions instituted among sub-populations identified here as having the highest HRI burden may result in the greatest reduction in HRI (and other heat-related outcomes). However, further analysis will be required to: clarify the relationship between HRI, race/ethnicity, and SES among Florida residents; identify additional susceptibility factors using or creating other data sources; and understand how all the susceptibility factors may work in conjunction with each other to increase an individual’s or community’s HRI risk.

## Figures and Tables

**Table 1:** Description of codes used to define work-relatedness within the morbidity data.

ICD-9-CM	Definition
-	Principal payer is workers’ compensation
E000.0	Civilian activity done for income or pay
E000.1	Military activity
E800-E807	Railway accident among railway employee (4th digit = 0)
E830-E838	Water transport accident among crew, dockers and stevedores (4th digit = 2 or 6)
E840-E845	Air and space transport accidents among crew and ground crew (4th digit = 2 or 8)
E846	Accidents involving powered vehicles used solely within the buildings and premises of industrial or commercial establishment
E849.1	Place of occurrence: farm building/land under cultivation
E849.2	Place of occurrence: mine or quarry
E849.3	Place of occurrence: industrial place and premises

**Table 2:** Distribution of non-work-related HRI among Florida residents by selected characteristics (2005–2012).

Characteristic	Florida Residents	HRI ED Visits			HRI Hospitalizations			HRI Deaths		
	N (%)	N (%)	Rate *	Rate Ratio (95% CI)	N (%)	Rate *	Rate Ratio (95% CI)	N (%)	Rate *	Rate Ratio (95% CI)
Gender										
Male	72,742,430 (48.9)	17,405 (72.6)	47.85	2.77 (2.69, 2.85)	3807 (79)	10.47	3.94 (3.68, 4.23)	94 (67.6)	0.26	2.18 (1.53, 3.11)
Female	76,018,379 (51.1)	6576 (27.4)	17.30	Reference	1009 (21)	2.65	Reference	45 (32.4)	0.12	Reference
Race ^†^										
White	117,671,057 (79.1)	17,511 (73.5)	29.76	Reference	3649 (76.5)	6.20	Reference	101 (72.7)	0.17	Reference
Black	24,114,183 (16.2)	5129 (21.5)	42.54	1.43 (1.39, 1.47)	870 (18.2)	7.22	1.16 (1.08, 1.25)	36 (25.9)	0.30	1.74 (1.19, 2.54)
Other	6,975,569 (4.7)	1182 (5)	33.89	1.14 (1.07, 1.21)	253 (5.3)	7.25	1.17 (1.03, 1.33)	2 (1.4)	0.06	0.33 (0.08, 1.35)
Ethnicity ^‡^										
Non-Hispanic	116,393,541 (78.2)	21,033 (88.6)	36.14	Reference	4194 (88.3)	7.21	Reference	124 (89.2)	0.21	Reference
Hispanic	32,367,268 (21.8)	2716 (11.4)	16.78	0.46 (0.45, 0.48)	557 (11.7)	3.44	0.48 (0.44, 0.52)	15 (10.8)	0.09	0.44 (0.25, 0.74)
Rural/Urban										
Urban	139,458,520 (93.7)	21,636 (90.2)	31.03	Reference	4379 (90.9)	6.28	Reference	127 (91.4)	0.18	Reference
Rural	9,302,289 (6.3)	2345 (9.8)	50.42	1.62 (1.56, 1.70)	437 (9.1)	9.40	1.50 (1.36, 1.65)	12 (8.6)	0.26	1.42 (0.78, 2.56)

* Crude rate per 100,000 person-years; ^†^ Missing race: ED = 159, Hospital = 44; ^‡^ Missing Ethnicity: ED = 232, Hospital = 65.

**Table 3:** Distribution of work-related HRI among Florida resident by selected characteristics (2005–2012).

Characteristic	Florida Residents	HRI ED Visits			HRI Hospitalizations			HRI Deaths		
	N (%)	N (%)	Rate *	Rate Ratio (95% CI)	N (%)	Rate *	Rate Ratio (95% CI)	N (%)	Rate *	Rate Ratio (95% CI)
Gender										
Male	35,690,570 (52.7)	2586 (86.8)	14.49	5.91 (5.32, 6.58)	396 (95.4)	2.22	18.73 (11.82, 29.69)	22 (95.7)	0.12	19.77 (2.67, 146.71)
Female	32,080,314 (47.3)	393 (13.2)	2.45	Reference	19 (4.6)	0.12	Reference	1 (4.3)	0.01	Reference
Race ^†^										
White	55,638,157 (82.1)	2348 (79.3)	8.44	Reference	298 (72)	1.07	Reference	16 (69.6)	0.06	Reference
Black	9,547,987 (14.1)	474 (16)	9.93	1.18 (1.07, 1.3)	88 (21.3)	1.84	1.72 (1.36, 2.18)	7 (30.4)	0.15	2.55 (1.05, 6.2)
Other	2,584,739 (3.8)	138 (4.7)	10.68	1.27 (1.07, 1.5)	28 (6.8)	2.17	2.02 (1.37, 2.98)	(0)	0.00	-
Ethnicity ^‡^										
Non-Hispanic	53,220,195 (78.5)	2560 (86.8)	9.62	Reference	324 (78.5)	1.22	Reference	14 (60.9)	0.05	Reference
Hispanic	14,550,689 (21.5)	389 (13.2)	5.35	0.56 (0.5, 0.62)	89 (21.5)	1.22	1.00 (0.79, 1.27)	9 (39.1)	0.12	2.35 (1.02, 5.43)
Rural/Urban										
Urban	62,309,645 (93.7)	2613 (87.7)	8.39	Reference	366 (88.2)	1.17	Reference	21 (91.3)	0.07	Reference
Rural	3,479,229 (6.3)	366 (12.3)	21.04	2.51 (2.25, 2.8)	49 (11.8)	2.82	2.4 (1.78, 3.23)	2 (8.7)	0.11	1.71 (0.40, 7.27)

* Crude rate per 100,000 person-years; ^†^ Missing race: ED = 19, Hospital = 1; ^‡^ Missing Ethnicity: ED =30, Hospital = 2.

## Supplementary Materials

The following are available online at www.mdpi.com/1660-4601/13/6/551/s1, Figure S1: County-specific counts of HRI ED visits and hospitalizations among Florida residents during the warm season (2005–2012): (A) non-work-related HRI ED visits; (B) work-related HRI ED visits; (C) Non-work-related HRI hospitalizations; (D) work-related HRI hospitalizations.

## Abbreviations

The following abbreviations are used in this manuscript:

ACS	American Community Survey
CDC	Centers for Disease Control and Prevention
CI	Confidence interval
CPS	Current Population Survey
Ecode	External cause of injury
ED	Emergency Department
FDOH	Florida Department of Health
ICD-9-CM	International Classification of Diseases, Ninth Revision, Clinical Modification
ICD-10	International Classification of Diseases, Tenth Revision
HRI	Heat-related illness
LOS	Length of stay
RR	Rate ratio
SE	Standard error
SES	Socio-economic status
U.S.	United States
