# Supplementary material for: A Comprehensive Evaluation of the Burden of Heat-Related Illness and Death within the Florida Population
Source: Int J Environ Res Public Health. 2016 May 31;13(6):551. doi: 10.3390/ijerph13060551 (PMC4924008; doi:10.3390/ijerph13060551)
Supplement: Supplementary file 1 [file ijerph-13-00551-s001.pdf]

Supplementary Materials: A Comprehensive Evaluation of the Burden of Heat-Related Illness and Death within the Florida Population

Laurel Harduar Morano, Sharon Watkins and Kristina Kintziger

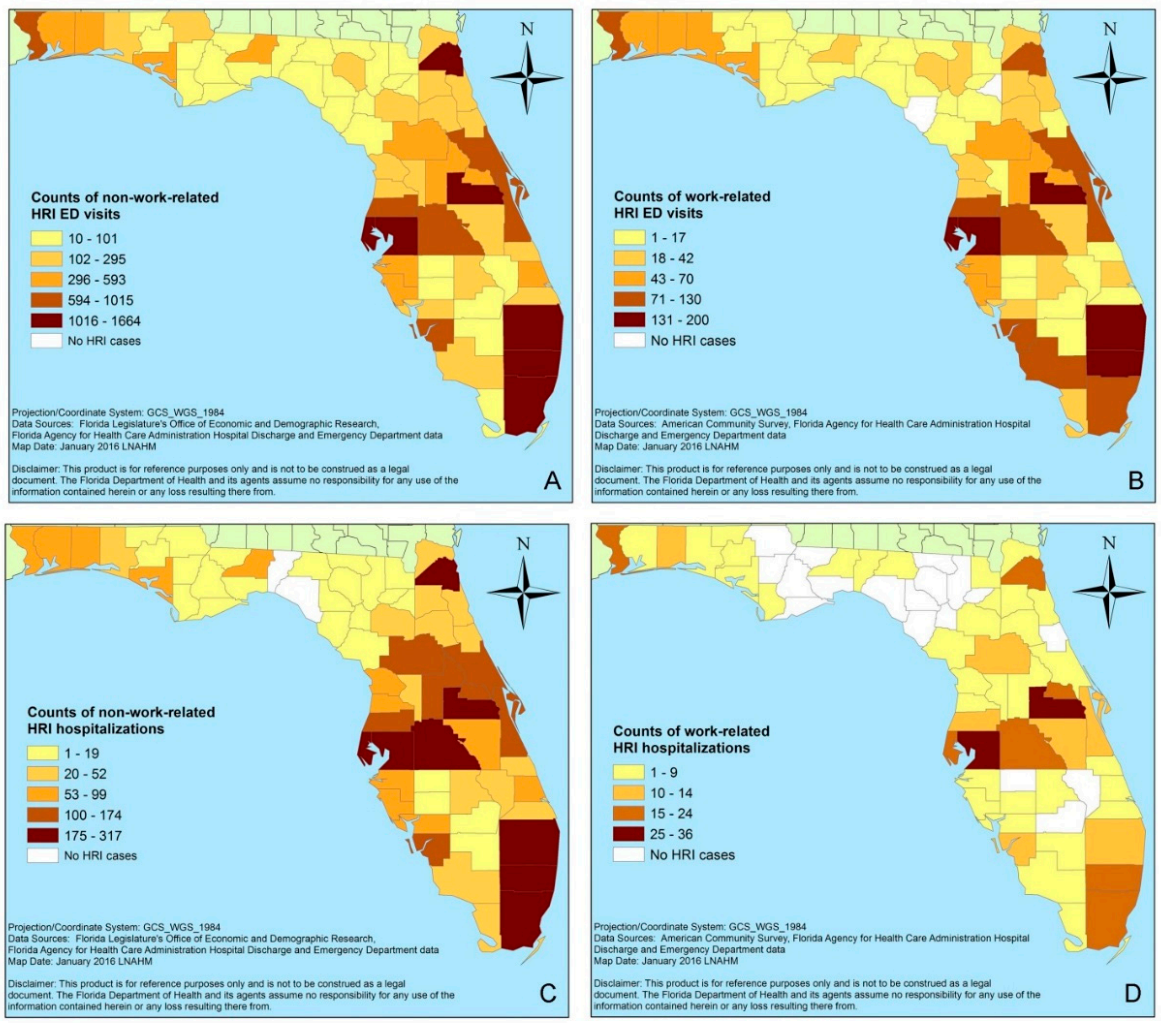

Figure S1. County-specific counts of HRI ED visits and hospitalizations among Florida residents during the warm season (2005–2012): (**A**) non-work-related HRI ED visits; (**B**) work-related HRI ED visits; (**C**) Non-work-related HRI hospitalizations; (**D**) work-related HRI hospitalizations.

© 2016 by the authors; licensee MDPI, Basel, Switzerland. This article is an open access article distributed under the terms and conditions of the Creative Commons by Attribution (CC-BY) license (http://creativecommons.org/licenses/by/4.0/).
